# Recombinant protein EBI3 attenuates *Clonorchis sinensis*-induced liver fibrosis by inhibiting hepatic stellate cell activation in mice

**DOI:** 10.1186/s13071-023-05863-5

**Published:** 2023-07-21

**Authors:** Lei Zhao, Jia Li, Gang Mo, Deping Cao, Chun Li, Guoyang Huang, Liping Jiang, Gen Chen, Hongbing Yao, Xiaohong Peng

**Affiliations:** 1 Guangxi University Key Laboratory of Pathogenic Biology, Guilin Medical University, Guilin, Guangxi People’s Republic of China; 2 Guangxi Key Laboratory of Molecular Medicine in Liver Injury and Repair, The Affiliated Hospital of Guilin Medical University, Guilin, Guangxi People’s Republic of China; 3 Second Affiliated Hospital of Guilin Medical University, Guilin, Guangxi People’s Republic of China

**Keywords:** Liver fibrosis, *Clonorchis sinensis*, EBI3, Hepatic stellate cell

## Abstract

**Background:**

Chronic infection with *Clonorchis sinensis* can cause hepatobiliary fibrosis and even lead to hepatobiliary carcinoma. Epstein-Barr virus-induced gene 3 protein (EBI3) is a subunit of interleukin 35, which can regulate inflammatory response and the occurrence of fibrotic diseases. Previous studies have reported that the expression of EBI3 in the serum of patients with liver cirrhosis is reduced. The present study aims to investigate the biological effects of EBI3 on liver fibrosis caused by *C. sinensis* and the underlying molecular mechanisms.

**Methods:**

We first established a mouse model of liver fibrosis induced by *C. sinensis* infection and then measured the serum expression of EBI3 during the inflammatory and fibrotic phase. GO (Gene Ontology) and KEGG (Kyoto Encyclopedia of Genes and Genomes) pathway analyses were performed to investigate the potential role of EBI3 in liver fibrosis by regulating the extracellular matrix structural constituent and collagen catabolic process. Recombinant protein EBI3 (rEBI3) was added to hepatic stellate cells (HSCs) in vitro with *C. sinensis* antigen to explore its function. Finally, the therapeutic effect of rEBI3 was verified by intravenous injection into *C. sinensis*-infected mice.

**Results:**

The results showed that the serum expression of EBI3 increased in the inflammatory response phase but decreased in the fibrotic phase. The excretory-secretory products of *C. sinensis* (Cs.ESP) were able to stimulate HSC activation, while rEBI3 reduced the activation of HSCs induced by Cs.ESP. Also, the protein expression of gp130 and downstream protein expressions of JAK1, p-JAK1, STAT3 and p-STAT3 in HSCs were increased after rEBI3 incubation. Finally, intravenously injected rEBI3 inhibited hepatic epithelial-mesenchymal transition in *C. sinensis*-infected mice by inhibiting HSC activation and reducing liver injury.

**Conclusion:**

This study confirms that rEBI3 can attenuate *C. sinensis-*induced liver fibrosis by inhibiting HSC activation and may be one of the potential treatments for liver fibrosis.

**Graphical Abstract:**

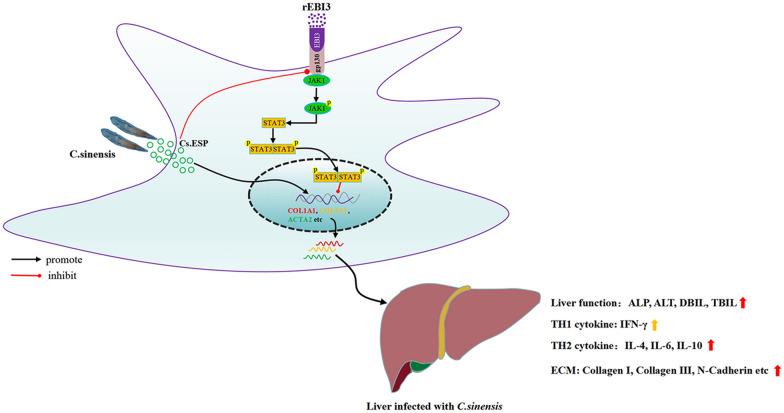

**Supplementary Information:**

The online version contains supplementary material available at 10.1186/s13071-023-05863-5.

## Background

Clonorchiasis, caused by infection with the trematode *Clonorchis sinensis*, is one of the most common food-borne trematodiases [1]. The consumption of raw or undercooked freshwater fish, such as *Pseudorasbora parva*, containing *C. sinensis* metacercariae is the key driving factor for the worldwide prevalence of clonorchiasis [2]. Clonorchiasis poses a significant public health issue to approximately 35 million individuals worldwide, with the highest incidence rates found in eastern Asian countries, such as China, Vietnam, Korea, Japan and parts of Russia [3, 4]. The adult parasites of *C. sinensis* are capable of inhabiting the biliary tract where they cause chronic inflammation, hyperplasia of the bile duct epithelium, bile duct fibrosis and, in severe cases, cholangiocarcinoma [5, 6].

Liver fibrosis is a wound-healing response that occurs in association with chronic liver injury. It is characterized by the excessive accumulation of extracellular matrix (ECM) elements such as collagen and fibronectin [7]. During the progress of liver fibrosis, hepatic stellate cells (HSCs), which are the pericytes of the liver residing in the space of Dissé between hepatocytes and endothelial cells, become the key effector cells. HSCs are known as the major source of collagen-producing myofibroblasts (MFBs) in fibrotic livers [8, 9]. In a quiescent state, HSCs store vitamin A-containing lipid droplets and express specific markers, such as desmin and lecithin retinol acyltransferase [10]. However, upon liver injury, HSCs are activated and differentiate into MFBs. They upregulate mesenchymal cell markers such as alpha-smooth muscle actin (α-SMA), collagen and N-cadherin, while losing their neural marker characteristics, ultimately leading to liver fibrosis [11].

Epstein-Barr virus-induced gene 3 protein (EBI3) is one of the subunits of interleukin (IL)-35 and IL-27, containing a signal peptide and two fibronectin type III domains [12]. Clinical studies have suggested that EBI3 expression is decreased in the serum of patients with liver cirrhosis [13, 14]. In vitro experiments have further revealed that EBI3 may reduce type I collagen expression when co-cultured with fibroblasts and alleviate pulmonary fibrosis induced by bleomycin by suppressing signal transducer and activator of transcription (STAT3) DNA binding [15, 16]. Notably, the supernatant of cultured HSCs can stimulate the generation of regulatory T cells (Treg cells), which produce IL-10 that in turn reduces the expression of α-SMA and type I collagen, thereby inhibiting HSC activation [17–19]. However, it remains unknown whether EBI3, a downstream target of Foxp3^+^ Treg cells [20], plays a role in mediating HSCs activation and regulating the development of liver fibrosis.

The aim of this study was to elucidate the biological effects of EBI3 on liver fibrosis induced by *C. sinensis* infection. To accomplish this, we established a mouse model of liver fibrosis using high-dose *C. sinensis* infection. We investigated the relationship between the expression of EBI3 and the expression of liver fibrosis markers and HSC activation markers. Moreover, we explored the potential of recombinant EBI3 (rEBI3) in inhibiting HSC activation and reducing liver fibrosis induced by *C. sinensis *in vitro and in vivo. Our data suggest that rEBI3 can effectively attenuate epithelial-mesenchymal transition (EMT) in the liver by inhibiting HSC activation, leading to the alleviation of liver fibrosis. These findings provide valuable insights for future research and the treatment of liver fibrosis.

## Methods

### Mice and metacercariae

Female BALB/c mice aged 6–8 weeks and weighing 18–22 g were obtained from the Hunan SJA Laboratory Animal Co., Ltd (Hunan, China). All animal studies were conducted in accordance with the guidelines and regulations of the Committee on Ethics of Animal Experiments of Guilin Medical University (GLMC202003199).

Fish infected with *C. sinensis* were purchased from Hengxian County, Guangxi, China. To obtain metacercariae, the fish were digested with hydrochloric acid following previously published protocols [21]. The resulting metacercariae were resuspended in 200 μl of normal saline (NS; 0.85% NaCl solution) to be used for oral gavage. The study protocol was reviewed and approved by the Committee on Ethics of Animal Experiments, Guilin Medical University (GLMC202003199).

### *Clonorchis sinensis* infection

To establish the liver fibrosis model, the group of mice designated as the infected group were administered 150 *C. sinensis* metacercariae through oral gavage; the group of mice designated as the control group received 200 μl of NS. Livers and serum were harvested at 1, 2, 4, 8 and 16 weeks after infection. The removed liver tissue was first weighed, following which part of the liver tissue was frozen at − 80 °C to be used in future western blot and PCR experiments, and the remaining part was immediately fixed and used for staining and histology studies. Serum was stored at − 80 °C for the cytokine assay.

### Functional enrichment analysis

The Genomic Spatial Event database for gene sequencing (GSE89147, GSE55747) for normal and fibrotic mouse tissue was downloaded from the GEO database (https://www.ncbi.nlm.nih.gov/geo/), with the official gene symbol selected as the identifier and mouse species selected. Pearson-correlation testing was performed using the R programming language with EBI3 as the target gene to obtain the EBI3-related gene set. The EBI3-related gene set was then imported into the DAVID portal website (https://david.ncifcrf.gov/home.jsp) to perform Gene Ontology (GO) analysis and Kyoto Encyclopedia of Genes and Genomes (KEGG) pathway analysis. The R package ClusterProfiler (Additional files 1) was used to generate images, and the top five results were displayed in the ascending order of the Benjamini procedure.

### Primary hepatic stellate cell isolation

Primary HSCs (pHSCs) were isolated using a two-step perfusion protocol as described previously [22]. Briefly, the liver was perfused in situ with an ethylene glycol tetraacetic acid (EGTA) solution heated to 37 °C, followed by sequential perfusion with Pronase E (Cat. No. P8360; 0.4 mg/ml; Solarbio, Beijing, China) for 5 min and Collagenase IV (Cat. No. C8160; 0.04%; Solarbio) for 7 min. The liver was then dissected, transferred into 50-ml Falcon tubes and digested with a buffer containing 0.4 mg/ml Pronase E, 0.04% Collagenase IV and 0.0125 mg/ml DNase I (Cat. No. D8071; Solarbio) in a 37 °C water bath with gentle agitation for 20 min. The resulting digested liver cells were passed through a 70-μm cell strainer and centrifuged at 580 *g* for 10 min, following which the liver cells were washed twice with Gey’s balanced salt solution (GBSS)/B buffer and resuspended in 16 ml GBSS/B buffer. Finally, the liver cells were subjected to density gradient separation with 9.69% Nycodenz (Histodenz) solution (Prod. No. 18003; Serumwerk Bernburg AG, Bernburg, Germany) and centrifuged at 1380 *g* for 17 min at 4 °C without brake to enrich HSCs. Cell viability was determined by using trypan blue staining, and cell purity was determined by using oil red O staining and indirect immunofluorescence detection of desmin (Additional file 1: Fig. S1A).

### In vitro rEBI3 treatment experiments

For in vitro experiments, *C. sinensis* excretory-secretory products (Cs.ESP) and *C. sinensis* adult crude antigen (Cs.CA) were prepared according to previously described methods [23, 24]. HSCs isolated from naive BALB/c mice were cultured in complete medium for 36 h and then starved for 12 h, following which, Cs.ESP (50 μg/ml) or Cs.CA (50 μg/ml) was added and the cells co-cultured for 12 h. Subsequently, rEBI3 was added to the co-cultured cells at a concentration of 10 ng/ml, and co-culture was continued for 48 h. Afterward, the supernatants and HSCs were collected for further analyses.

The rEBI3 used in this study was a synthetic peptide with a gene sequence of CGAATTTCTACACTGAAACAGCTCTCGTGGCCTAAGCCAGCCCAGAGTGCAATGCCATGCTTCTCGGTATCCCGTGGCCGTGGACTGCTCCTGGACTCCTCTCCAGGCTCCCAACTCCACCAGATCCACGTCCTTCATTGCCACTTACAGGCTCGGTGTGGCCACCCAGCAGCAGAGCCAGCCCTGCCTACAACGGAGCCCCCAGGCCTCCCGATGCAC, provided by Cloud-Clone Corp. (formerly USCN Life Science Inc.) in Wuhan, China (Cat: RPD206Mu01) (Additional file 1: Fig. S1B).

### In vivo rEBI3 treatment experiment

Mice were divided into five groups, each consisting of six mice. The five groups included: (i) a blank group (without any treatment); (ii) NS-NS group (NS oral gavage and NS tail vein injection); (iii), NS-rEBI3 group (NS oral gavage and rEBI3 tail vein injection); (iv) 150M-NS group (150 metacercariae oral gavage and NS tail vein injection); and (v) 150M-rEBI3 group (150 metacercariae oral gavage and rEBI3 tail vein injection). From 3 days post-infection onwards, the mice received intravenous injections of 3.5 μg rEBI3 (dissolved in 100 μl NS) or of 100 μl NS at 7-day intervals until day 24 post-infection. The mice were then sacrificed, and liver and serum samples were harvested at 28 days after infection.

### Immunofluorescence and cytokine analysis

For the immunofluorescence experiments, HSCs were washed 3 times with phosphate-buffered saline and fixed in 4% paraformaldehyde at room temperature for 10 min. Fixed cells were first incubated with an anti-Desmin antibody (Cat. No. A3736; ABclonal, Wuhan, China) at 4 °C overnight, and then incubated with CoraLite488-conjugated Goat Anti-Rabbit Immunoglobulin G (IgG) (H + L) (Cat. No. SA00013-2; Proteintech Group, Rosemont, IL, USA) for 1 h at 37 °C. DAPI stain was added to the incubated cells and incubation continued for 5 min. The images were acquired using an Olympus BX53 microscope (Olympus Corp., Tokyo, Japan).

The level of serum EBI3, collagen I and α-SMA levels were measured using commercially available enzyme-linked immunosorbent assay (ELISA) kits (Cat. Nos. MM-46375M1, MM-0993M1, MM-44558M1; MEIMIAN, Jiangsu, China), following the manufacturer’s standard protocols. The levels of cytokines interferon gamma (IFN-γ), IL-4, IL-6, IL-10 and tumor necrosis factor (TNF) were measured using a BD Cytometric Bead Array Kit (Cat. No. 560485; BD Biosciences, Franklin Lakes, NJ, USA) and analyzed on a FACSCanto flow cytometer (BD Biosciences).

### Histological and biochemical assessment

Liver samples were collected and embedded in paraffin as described previously [25], and 4-μm serial sections were stained with hematoxylin and eosin (H&E) and Masson’s trichrome staining and viewed under an Olympus BX53 microscope system (Olympus Corp.). For the quantification of Masson’s staining, Image J software (National Institutes of Health, Bethesda, MD, USA) was employed to measure the area of collagen fiber.

The serum levels of alanine aminotransferase (ALT), alkaline phosphatase (ALP), direct bilirubin (DBIL), total bilirubin (TBIL) and hepatic hydroxyproline (HYP) were measured by commercially available kits (Nanjing Jiancheng Bioengineering Institute, Nanjing, China) following the manufacturer’s standard protocols.

The fibrosis grade was determined by calculating the average integrated optical density of randomly selected six areas under microscope and by detecting of hydroxyproline content.

### Quantitative real-time PCR analysis

Total RNA was extracted from liver tissue samples using TRIzol reagent (Cat. No. 15596026; Invitrogen, Thermo Fisher Scientific, Waltham, MA, USA), as previously described [25]. The synthesis of complementary DNA (cDNA) was performed using the MonScriptTMRTIII All-in-One Mix with dsDNase Kit (Cat. No. MR0511; Monad, Guangzhou, China). Real-time PCR reactions were conducted using the MonAmpTM Fast SYBR® Green qPCR Mix Kit (Cat No. MQ20301S; Monad), following the manufacturer’s instructions. The fold changes in messenger RNA (mRNA) levels were calculated by the 2^−∆∆Ct^ method [26]. The primer sequences (Genecreate, Hubei, China) are listed in Additional file 1: Table S1.

### Western blot analysis

For the extraction of total proteins, liver tissue samples were homogenized with RIPA lysis buffer (Cat. No. PC101; Shanghai Epizyme Biomedical Technology Co., Ltd, Shanghai, China) containing 1% 100 mM protease inhibitor mixture (PMSF) (Cat. No. P6730; Solarbio) and Phosphatase Inhibitor Cocktail (Cat. No. BL615A; Biosharo, China). The protein concentrations were determined using the BCA Protein Assay Kit (Cat No. P0010s; Beyotime Biotechnology Co. Ltd, Shanghai, China). Protein samples of 50 μg per lane were separated using in an 8%/10% sodium dodecyl sulfate-polyacrylamide gel electrophoresis gel and transferred onto polyvinylidene fluoride membranes. The membranes were blocked with 5% non-fat milk for 1 h or with the QuickBlock Western Kit (Cat. No. P0252; Beyotime Biotechnology Co. Ltd) for 30 min at room temperature. The blots were then incubated with different antibodies at 4 °C overnight. The primary antibodies are listed in Additional file 1: Table S2. The secondary anti-rabbit (ABclonal) IgG antibody was incubated for 1 h at room temperature. The ECL Detection kit (Cat. No. SQ201; Shanghai Epizyme Biomedical Technology Co., Ltd) was employed to develop the blots, and immunoreactive bands were visualized with chemiluminescence detection systems (ChemiDoc XRS+; Bio-Rad Technologies, Hercules, CA, USA).

### Statistical analysis

All values are presented as the mean ± standard deviation (SD). Unpaired t-tests and one-way analysis of variance (ANOVA) were used to assess the differences between groups using SPSS version 26.0 software (SPSS, IBM Corp., Armonk, NY, USA) and GraphPad Prism 9.0 software (GraphPad Software Inc., San Diego, CA, USA). A *P*-value < 0.05 was considered to indicate statistical significance.

## Results

### Collagen Content in the liver of mice increased with prolonged infection of *C. sinensis*

**Fig. 1:**
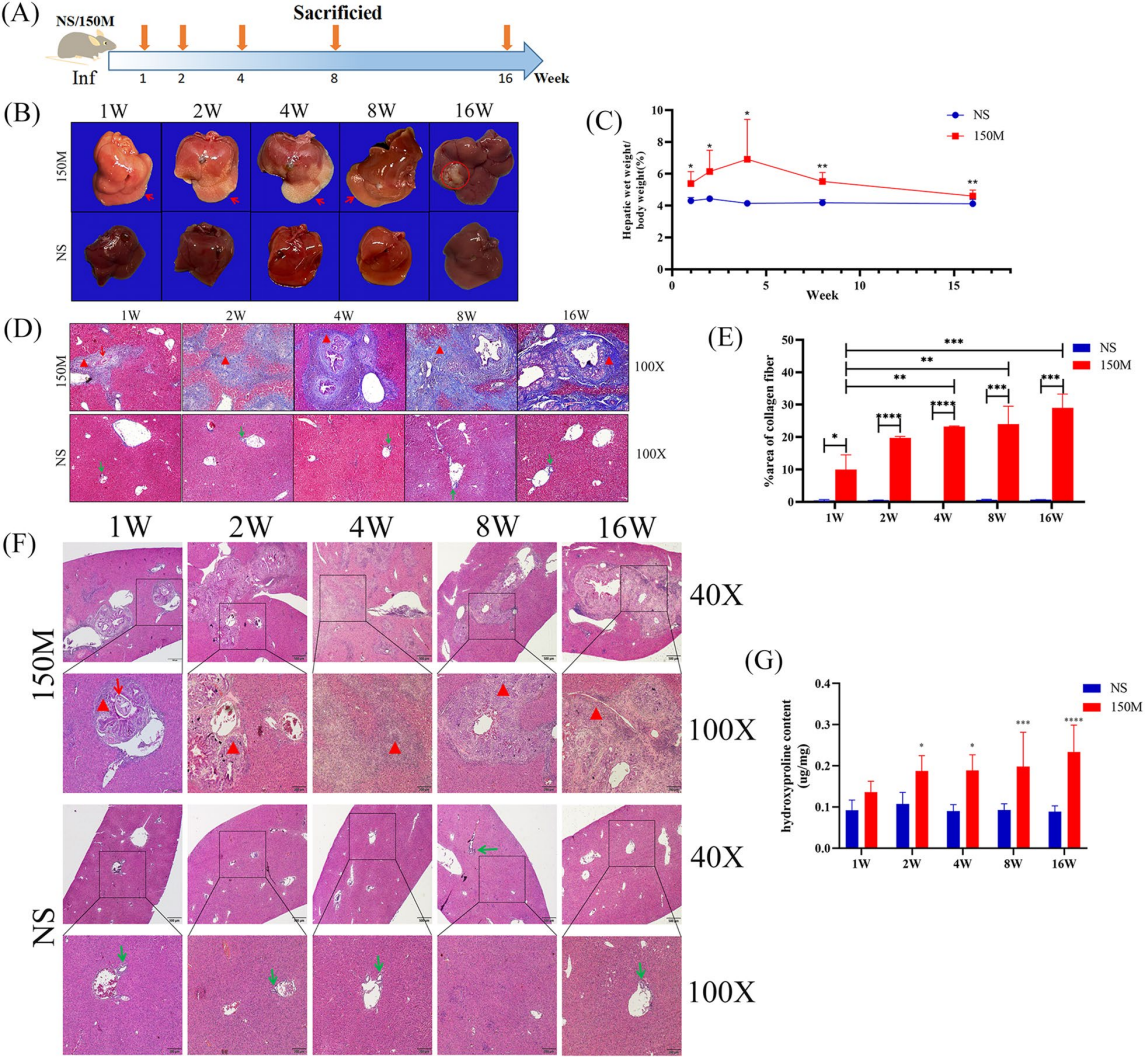
Morphological changes of mice livers after *Clonorchis sinensis* infection. **a** Study design. Female BALB/c mice were orally gavaged with 150 *C. sinensis* metacercariae (150M) or NS at the indicated time point. Mice were sacrificed at 1 week, 2 weeks, 4 weeks, 8 weeks and 16 weeks after the infection. **b** Morphological changes in mouse liver. Red arrows indicate diseased tissue; red circle indicates liver fibrosis region.** c** Ratio of liver weight to body weight of mice (%). **d** Masson’s trichrome staining of mice livers. Red arrows indicate the worms; red triangles indicate the fibrosis area; green arrows indicate the hepatobiliary area. **e** Collagen fiber expression level in the infected and normal liver tissues visualized used Image J software. **f** HE staining of mice livers. The red arrows indicate the worms, the red triangle indicates the inflammatory cell infiltration area and green arrows indicate the hepatobiliary area. **g** Hydroxyproline content in the infected and normal liver tissues.** c**,** e**,** g** Data are presented as the mean ± SD, representing one of two independent experiments, with 6 mice per group, and compared by one-way ANOVA. Asterisks denote significant difference at *P < 0.05, **P < 0.01, ***P < 0.001 and ****P < 0.0001. Abbreviations: ANOVA, Analysis of variance; HE, hematoxylin and eosin; NS, normal saline; SD, standard deviation; W, week

We reported previously that high-dose *C. sinensis* infection could be used as a better model of liver fibrosis in mice than low-dose infection [27]. In the present study, we first detected the dynamics of liver morphology changes following infection with 150 *C. sinensis* metacercariae. The livers of the infected mice showed a significant increase in size and severe swelling within the first 4 weeks after infection. At week 16 post-infection, liver morphology was comparable to that of uninfected mice (Fig. 1b, c). HE staining of the liver showed inflammatory cell infiltration around the parasitized liver bile duct in the first 4 weeks post-infection, which gradually decreased in intensity after 8 weeks and further decreased after 16 weeks post-infection (Fig. 1f). Masson’s staining showed that collagen deposition around the hepatobiliary duct gradually progressed with increasing duration of infection (Fig. 1d). The quantification of Masson’s staining and the detection of hydroxyproline content showed that hepatic fibrosis was aggravated with prolongation of infection time (Fig. 1e, g).

### Serum EBI3 increased during inflammatory phase but declined in fibrogenesis phase

**Fig. 2:**
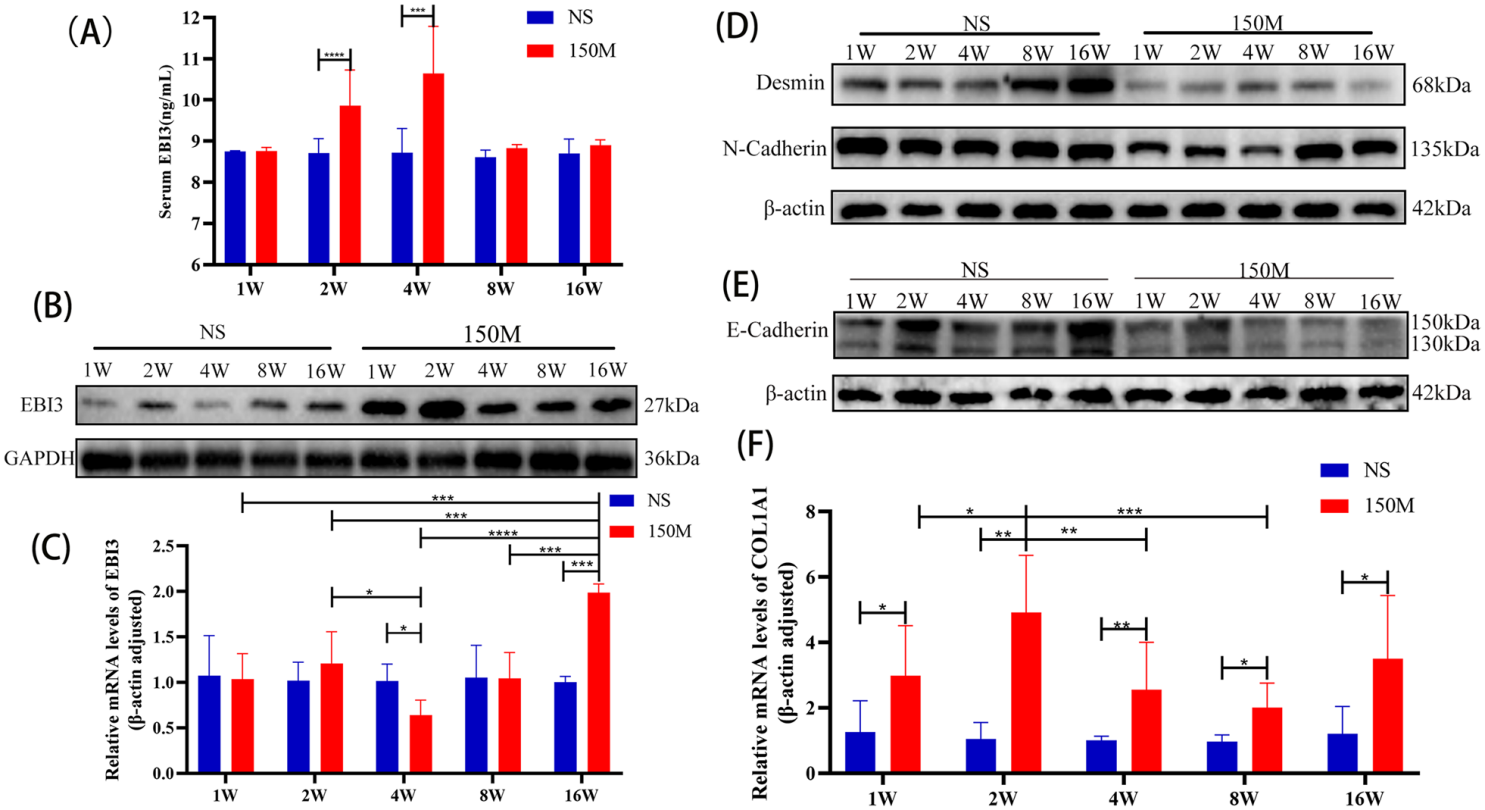
Increased expression of EBI3 in liver fibrosis. **a** Serum EBI3 levels at the second and third week of *C. sinensis* infection. Compared with the NS group. **b**, **c** Expression profiles of EBI3 in liver of mice orally gavaged with NS or *C. sinensis* metacercariae: **b** expression of EBI3 protein in different periods after infection, **c** relative expression of EBI3 mRNA in different periods after infection. **d**, **e** Relative expression of desmin, N-cadherin and E-cadherin protein in different periods after infection. **f** Relative expression of COL1A1 mRNA in different periods after infection.** a** Data are presented as mean ± SD and were analyzed by the unpaired t-test (two-tailed);** c**,** f** data are presented as the mean ± SD, representing one of two independent experiments, with 6 mice per group, and compared by one-way ANOVA. Asterisks denote significant difference at *P < 0.05, **P < 0.01, ***P < 0.001 and ****P < 0.0001. Abbreviations: ANOVA, Analysis of variance; COL1A1, collagen type I alpha 1; EB13, Epstein-Barr virus-induced gene 3; GAPDH, glyceraldehyde 3-phosphate dehydrogenase; mRNA, messenger RNA; NS, normal saline; SD, standard deviation; W, week; 150M, 150 *C. sinensis* metacercariae

To analyze the dynamics of EBI3 expression during *C. sinensis* infection, we first detected EBI3 expression in the serum of experimentally infected mice. Compared with the NS group, the serum EBI3 content in the infection group had increased at week 2, subsequently peaking at week 4 (Fig. 2a) and declining at week 8. The EBI3 protein level in the liver of the experimentally infected mice increased significantly compared with the NS group, peaking at week 2 and then declining gradually (Fig. 2b; Additional file 1: Fig. S2A). Interestingly, during the first 2 weeks post-infection, there was no significant change in the hepatic EBI3 mRNA transcript level of the infected mice compared with the NS group; however, the hepatic EBI3 mRNA transcript level of infected mice decreased significantly at week 4, following which time it gradually increased, reaching significantly higher levels than in the NS group at week 16 (Fig. 2c).

As a marker of HSC, liver desmin protein expression increased with age in the NS group but decreased in the infected mice. To further explore the process of liver fibrosis in mice, we detected E-cadherin and N-cadherin in the liver and found that in the infected mice, the expression of E-cadherin decreased with the prolongation of infection time, while the expression of N-cadherin decreased in the first 4 weeks and then increased at weeks 8 and 16 post-infection (Fig. 2d, e; Additional file 1: Fig. S2B–D). Quantitative real-time PCR also showed that the transcript level of COL1A1 was higher in the liver of the infected mice than in the liver of mice in the NS group (Fig. 2f).

### Predicting EBI3 regulates collagen fibril organization response through ECM structural constituents

**Fig. 3:**
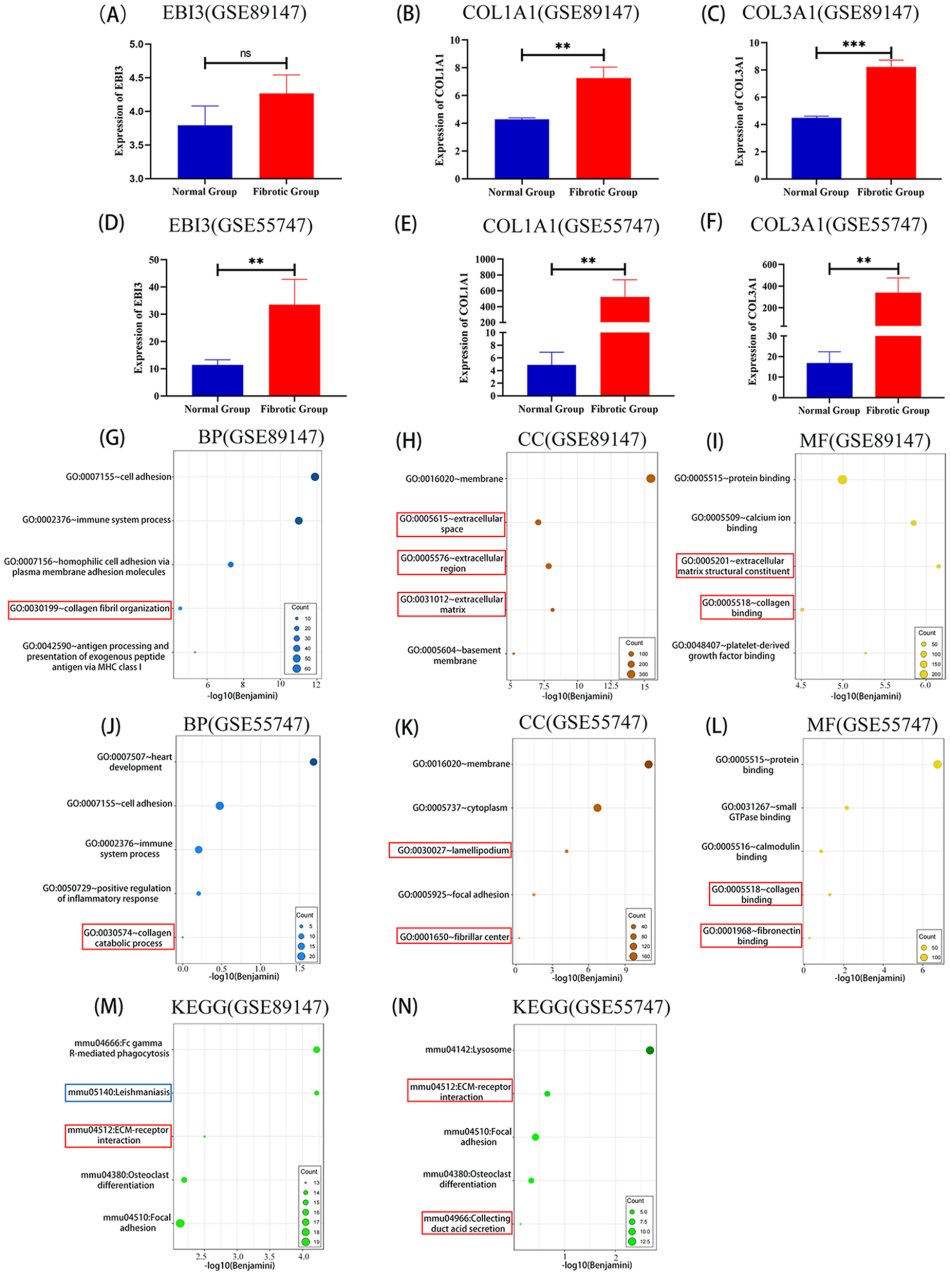
EBI3 is closely associated with collagen fibril organization process regulation in liver fibrosis. **a–f** EBI3, COL1A1 and COL3A1 mRNA expression assay in the normal and fibrotic liver from mice. **g–l** BP, CC and MF are mostly related to EBI3 in the Gene Expression Omnibus (GEO; GSE89147, GSE55747) database. **m, n** KEGG pathway analysis of EBI3 in the GEO databases. The data are presented as mean ± SD and were analyzed by unpaired the t-test (two-tailed). Asterisks indicate a significant difference at **P < 0.01 and ***P < 0.001. Abbreviations: BP, Biological processes; CC, cellular components; COL1A1, collagen type I alpha 1; COL3A1, collagen type III alpha 1; EB13, Epstein-Barr virus-induced gene 3; GO, Gene Ontology; GSE, Genomic Spatial Event database; KEGG, Kyoto Encyclopedia of Genes and Genomes; MF, molecular functions; mRNA, messenger RNA; SD, standard deviation

In order to further study the mechanism of EBI3 in the formation of liver fibrosis, we performed GO and KEGG pathway analysis to predict the function of EBI3. In the GSE89147 database, we found that collagen type I alpha 1 (COL1A1) and collagen type III alpha 1 (COL3A1) mRNA expressions were significantly higher in mice of the liver fibrosis group than in mice in the normal group and that the expression of EBI3 mRNA showed a trend of being higher in the liver fibrosis group than that in the normal group, although the difference was not significant (Fig.3a-c). To investigate the biological functions related to EBI3, we screened the five genes most related to EBI3 based on Pearson correlation analysis (Cor > 0.7, *P* < 0.05, 628 genes were acquired) in the Gene Expression Omnibus (GEO) databases and performed GO and KEGG analyses based on the above gene sets. In the GEO database, biological processes most related to EBI3 included collagen fibril organization, cell adhesion and immune system process (Fig. 3g). Moreover, EBI3’s most related cellular components were the membrane, including ECM, the extracellular region and extracellular space (Fig. 3h). The molecular functions most related to EBI3 were ECM structural constituents, including calcium ion binding and collagen binding (Fig. 3i), while the signaling pathways most related to EBI3 included Fc gamma R-mediated phagocytosis and ECM-receptor interaction (Fig. 3m). In the GSE55747 database, we found that EBI3, COL1A1 and COL3A1 mRNA expressions in mice of the liver fibrosis group were also higher than those of the normal group (Fig.3d-f). The EBI3-related biological processes, cellular components, molecular functions and signaling pathway in the GSE89147 database were similar to those in the GSE55747 database (Fig. 3j–l, n). Therefore, we predict that EBI3 probably played an essential role in regulating ECM structural constituents and collagen catabolic processes.

### rEBI3 decreases the expression of related fibrin protein in HSCs by targeting JAK1 and STAT3

**Fig. 4:**
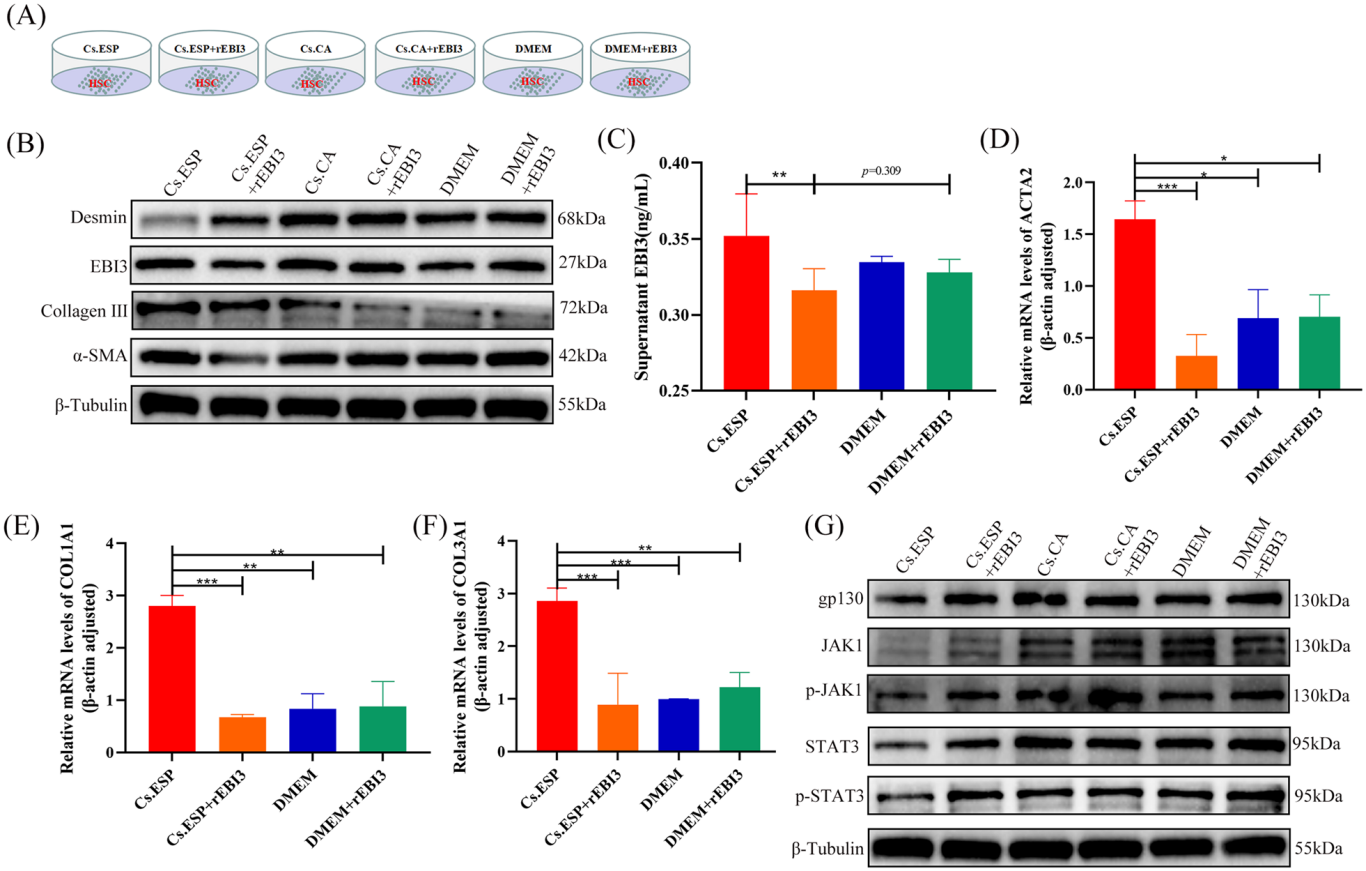
rEBI3 can inhibit the activation of HSC induced by Cs.ESP in vitro. **a** Design of HSC co-culture experiment. **b** Protein levels of EBI3, α-SMA, desmin, collagen I and collagen III in the co-cultured HSCs with Cs.ESP, Cs.ESP + rEBI3, Cs.CA, Cs.CA + rEBI3, DMEM and DMEM + rEBI3. **c** EBI3 expression in co-culture supernatant. **d–f** mRNA relative expression of ACTA2, COL1A1 and COL3A1. **g** Protein levels of gp130, JAK1, p-JAK1, STAT3 and p-STAT3 in the HSCs.** c**,** d**,** e**,** f** Data are presented as the mean ± SD, representing one of two independent experiments, with each experimental group making 3 multiple holes, and compared using the one-way ANOVA. Asterisks indicate a significant difference at *P < 0.05, **P < 0.01 and ***P < 0.001. Abbreviations: ACTA2, Actin alpha 2, smooth muscle; ANOVA, analysis of variance; Cs.ESP, *C. sinensis* excretory-secretory products; Cs.CA *C. sinensis* adult crude antigen; DMEM, Dulbecco's Modified Eagle Medium; HSC, hepatic stellate cell; gp130, beta receptor glycoprotein 130; mRNA, messenger RNA; (p)-JAK, (phosphorylated) Janus tyrosine kinase; (p)-STAT3, (phosphorylated)-signal transducer and activator of transcription 3; rEBI2, recombinant EBI3; α-SMA, alpha-smooth muscle actin; SD, standard deviation

The activation of HSCs is the main trigger of liver fibrosis. To explore the effect of EBI3 on HSC activation, we performed an in vitro experiment by culturing HSCs with excretory-secretory products of *C. sinensis* (Cs.ESP) or crude antigens of adult *C. sinensis* (Cs.CA) followed by rEBI3 treatment (Fig. 4a). The results showed that Cs.ESP could stimulate HSCs to express EBI3, while the expression of EBI3 was significantly reduced after rEBI3 treatment both in the culture supernatant and in the HSCs (Fig. 4b, c; Additional file 1: Fig. S1E). Meanwhile, desmin expression in HSCs was significantly decreased, while the mRNA transcription levels of COL1A1, COL3A1 and actin alpha 2, smooth muscle (ACTA2) were increased after Cs.ESP stimulation, indicating that HSCs could be activated by Cs.ESP (Fig. 4b, d–f; Additional file 1: Fig. S1F). However, compared with the Cs.ESP group, the expression of desmin in the Cs.ESP + rEBI3 group was increased, and the expression of α-SMA and collagen III was significantly decreased (Fig. 4b; Additional file 1: Fig. S1F–S1H). Moreover, the mRNA transcription levels of COL1A1, COL3A1 and ACTA2 in Cs.ESP + rEBI3 group were also decreased compared with those in the Cs.ESP group (Fig. 4d–f). Cs.CA showed no significant activation effect on HSCs (Fig. 4b; Additional file 1: Fig. S1G). In sum, the results indicate that rEBI3 could directly inhibit Cs.ESP-induced HSC activation and reduce the production of related ECM.

Subsequently, we explored the related signaling pathway proteins and observed a decrease in the expression of beta receptor glycoprotein 130 (gp130) and downstream proteins, including Janus kinase 1 (JAK1), phosphorylated JAK1 (p-JAK1), STAT3 and phosphorylated STAT3 (p-STAT3), after Cs.ESP stimulation (Cs.ESP line). However, the expression of these proteins significantly increased after rEBI3 treatment (Cs.ESP + rEBI3 line) (Fig. 4g; Additional file 1: Fig. S1I–M). These results indicate that rEBI3 could bind with gp130 and activate the JAK1/STAT3 pathway, ultimately inhibiting the production of the related fibrotic cytokines.

### rEBI3 inhibits liver epithelial-mesenchymal transition and reduces liver fibrosis in the infected mice by activating the JAK1/STAT3 pathway

**Fig. 5:**
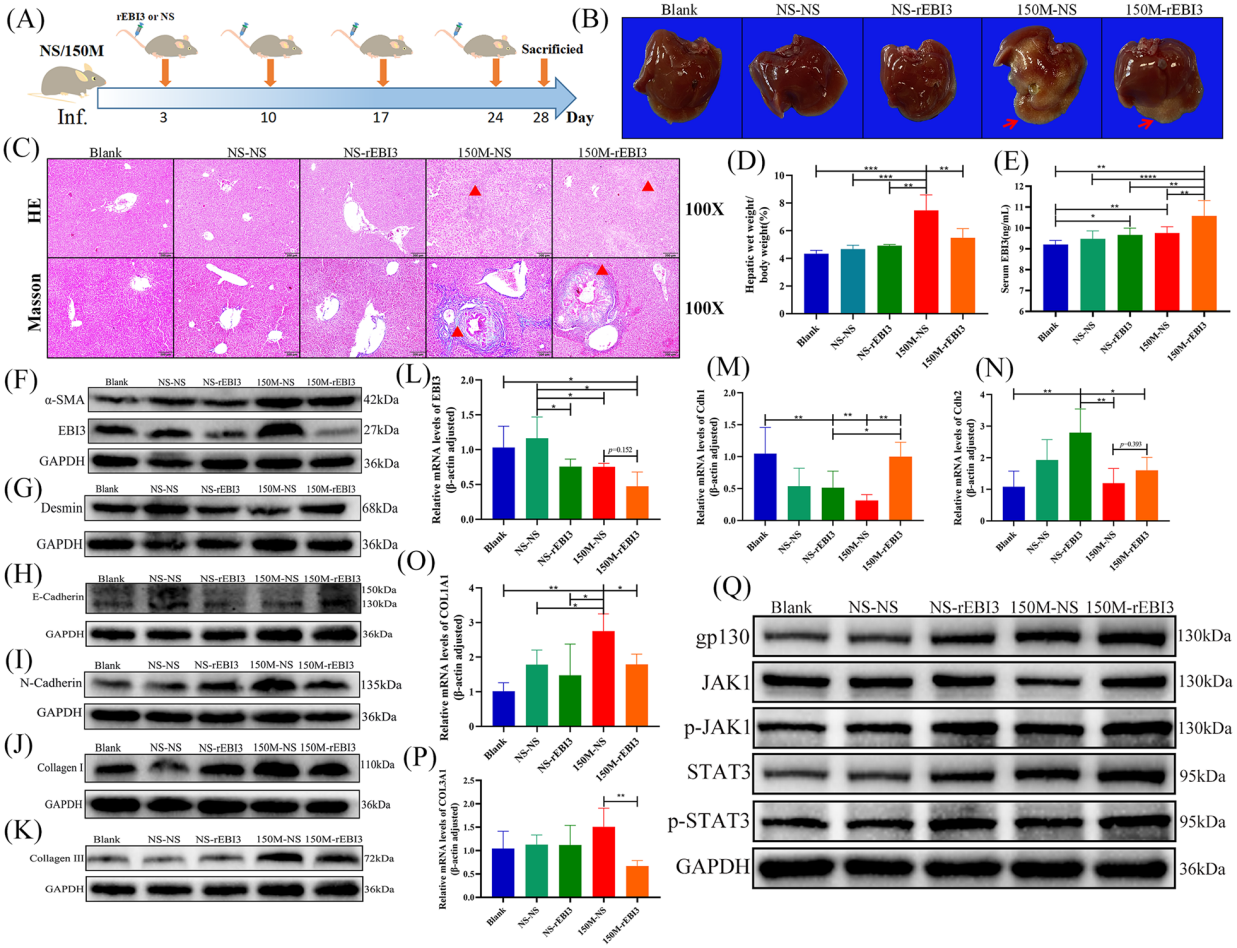
Tail vein injection of rEBI3 inhibits hepatic epithelial-mesenchymal transition in *C. sinensis*-infected mice. **a** The study design. Mice were injected with rEBI3 (3.5 μg/100 μl) or NS (100 μl) at the indicated time courses.** b** Morphological and pathological changes in mice livers. Red arrows indicate the diseased region.** c** HE and Masson’s trichrome staining of mice livers: red triangles indicate the inflammatory and fibrosis area.** d** Ratio of liver weight to body weight of mice (%).** e** Levels of serum EBI3 after injections of NS or rEBI3. Compared with the indicated groups. **f–k** Protein levels of α-SMA, EBI3, desmin, E-cadherin, N-cadherin, collagen I and collagen III in the livers of mice intravenously injected with NS or rEBI3. **l–p** EBI3, Cdh1, Cdh2, COL1A1 and COL3A1mRNA relative expression in the livers of mice intravenously injected with NS or rEBI3. **q** Protein levels of gp130, JAK1, p-JAK1, STAT3 and p-STAT3 in the livers of mice intravenously injected with NS or rEBI3. **d**,** e**,** l**,** m**,** n**,** o**,** p** Data are presented as the mean ± SD, representing 6 individually analyzed mice in each group, and compared using one-way ANOVA. Asterisks indicate a significant difference at *P < 0.05, **P < 0.01, ***P < 0.001 and ****P < 0.0001. Experimental groups: NS-NS, NS oral gavage and NS tail vein injection; NS-rEBI3, NS oral gavage and rEBI3 tail vein injection; 150M-NS, 150 metacercariae oral gavage and NS tail vein injection; 150M-rEBI3, 150 metacercariae oral gavage and rEBI3 tail vein injection. Abbreviations: Cdh1/2, cadherin 1/2; COL1A1, collagen type I alpha 1; COL3A1, collagen type III alpha 1; GAPDH, glyceraldehyde 3-phosphate dehydrogenase; gp130, beta receptor glycoprotein 130; mRNA, messenger RNA; NS, normal saline; (p)-JAK, (phosphorylated) Janus tyrosine kinase; (p)-STAT3, (phosphorylated)-signal transducer and activator of transcription 3; rEBI2, recombinant EBI3; α-SMA, alpha-smooth muscle actin; SD, standard deviation; 150M, 150 *C. sinensis* metacercariae

To verify whether rEBI3 has the same effect in vivo, we treated *C. sinensis*-infected mice with rEBI3 by tail vein injection (Fig. 5a) and found that the lesion area of mice in the 150M-rEBI3 group was significantly lower than that in mice of the of 150M-NS group (Fig. 5b). The HE and Masson’s staining results also showed that collagen deposition around the bile ducts in the liver and the liver wet weight were significantly reduced in the 150M-rEBI3 group (Fig. 5c, d). Next, we detected the serum EBI3 protein levels. The results showed that the serum EBI3 level in the NS-rEBI3, 150M-NS and 150M-rEBI3 groups was significantly increased, with mice in the 150M-rEBI3 group showing the highest EBI3 expression compared with the other groups (Fig. 5e). Subsequently, we detected the related liver proteins and mRNA transcription levels of mice. The results, which were consistent with those shown in Fig. 2c, revealed that the EBI3 protein in the 150M-NS group was significantly increased. Remarkably, the expression of EBI3 in the 150M-rEBI3 mice was significantly decreased (Fig. 5f; Additional file 1: Fig. S3B). Furthermore, the EBI3 mRNA transcription levels were decreased in both the 150M-NS and 150M-rEBI3 groups (Fig. 5l). Moreover, the expression of desmin in the 150M-rEBI3 mice was significantly increased compared to that in mice in the 150M-NS group (Fig. 5g; Additional file 1: Fig. S3C), indicating that the tail vein injection of rEBI3 could inhibit the activation of HSCs in *C. sinensis*-infected mice.

We further tested E-cadherin and N-Cadherin expression and found that the E-cadherin in the 150M-rEBI3 mice was increased compared to 150 M-NS mice, whereas N-cadherin decreased (Fig. 5h, i, m, n; Additional file 1: Fig. S3D–E), indicating that tail vein injection of rEBI3 protein could inhibit the EMT in the mouse liver. We also detected fibrosis-related proteins (α-SMA, collagen I, collagen III) and mRNA transcript levels of α-SMA, COL1A1 and COL3A1. These results showed that compared with the 150M-NS mice, the α-SMA, collagen I and collagen III proteins and ACTA2, COL1A1, COL3A1 mRNA transcript levels of 150M-rEBI3 mice were decreased (Fig. 5f, j, k, o, p; Additional file 1: Fig. S3A, F–H).

Finally, the expression of gp130 protein in mice in the NS-EBI3, 150M-NS and 150M-rEBI3 group was significantly increased. Compared with the 150M-NS group, the expression of JAK1, p-JAK1, STAT3 and p-STAT3 proteins in the 150M-rEBI3 group increased significantly (Fig. 5q; Additional file 1: Fig. S4A–S4E). These data suggest that tail vein injection of rEBI3 can inhibit liver EMT and reduce liver fibrosis in *C. sinensis*- infected mice by activating the JAK1/STAT3 pathway.

### rEBI3 reverses the biliary injuries and hepatic inflammatory responses caused by *C. sinensis*

**Fig. 6:**
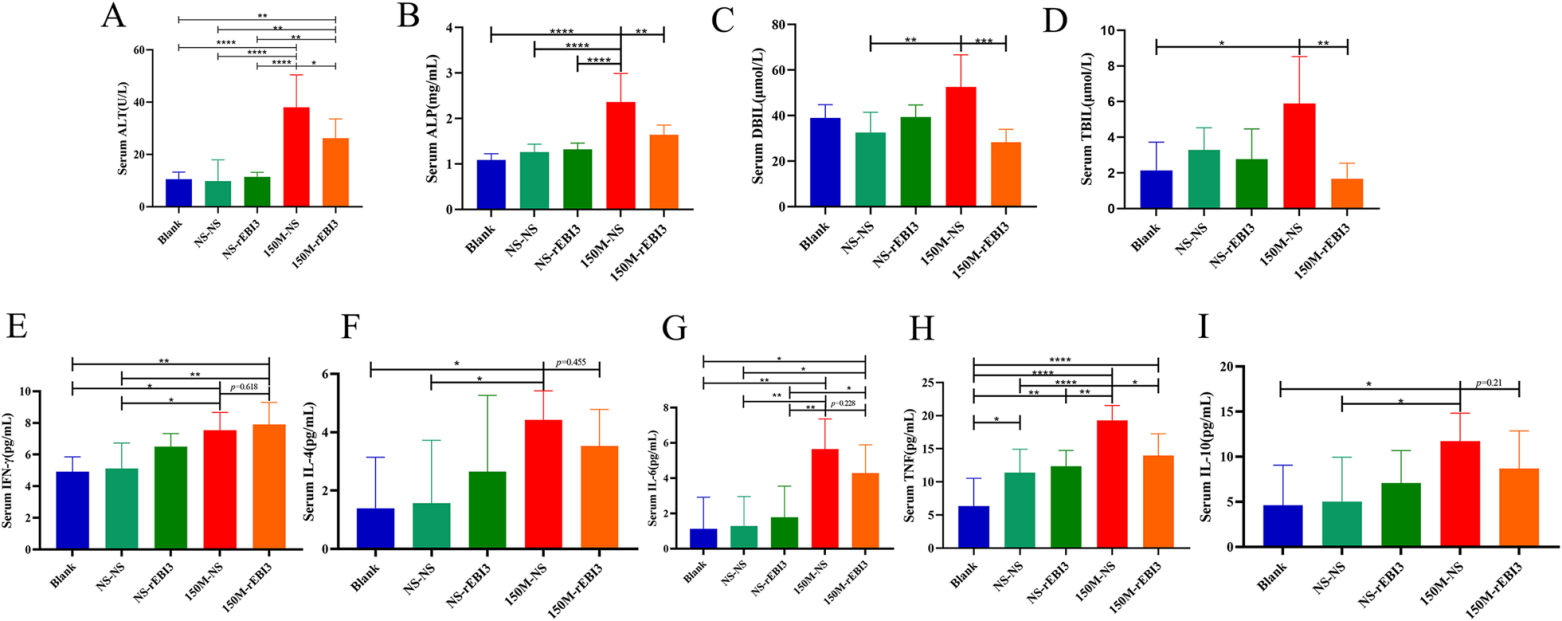
rEBI3 reduces liver injury in *C. sinensis* infected mice. **a**–**d** Serum levels of liver ALT, ALP, DBIL and TBIL. **e–i** Levels of IFN-γ, IL-4, IL-6, TNF and IL-10 in the livers of mice. Data are presented as the mean ± SD, representing 6 individually analyzed mice in each group, and compared with one-way ANOVA. Asterisks indicate a significant difference at *P < 0.05, **P < 0.01, ***P < 0.001 and ****P < 0.0001. Experimental groups: NS-NS, NS oral gavage and NS tail vein injection; NS-rEBI3, NS oral gavage and rEBI3 tail vein injection; 150M-NS, 150 metacercariae oral gavage and NS tail vein injection; 150M-rEBI3, 150 metacercariae oral gavage and rEBI3 tail vein injection. Abbreviations: ALP, Alkaline phosphatase; ALT, alanine aminotransferase; ANOVA, analysis of variance; DBIL, direct bilirubin; IFN, interferon; IL, interleukin; NS, normal saline; rEBI3, recombinant EBI3; TBIL, total bilirubin; TNF, tumor necrosis factor; 150M, 150 *C. sinensis* metacercariae

To understand the impact of rEBI3 treatment on liver function and the related immune response, liver enzyme levels, bile acids and cytokines were analyzed. Compared with the 150M-NS group, the serum levels of ALT, ALP, DBIL and TBIL in the 150M-EBI3 group were significantly lower (Fig. 6a–d). Moreover, the level of IFN-γ was increased (Fig. 6e), whereas IL-4, TNF, IL-6 and IL-10 levels were relatively decreased (Fig. 6f–i). These data suggested that tail vein injection of rEBI3 did not result in liver injury and may decrease the incidence of liver fibrosis by upregulating anti-fibrotic cytokine expression and downregulating pro-fibrotic cytokine expression.

## Discussion

*Clonorchis sinensis*, also known as liver fluke, can parasitize in the hepatobiliary duct and cause cholestasis, leading to chronic liver damage, liver fibrosis, liver cirrhosis and even cholangiocarcinoma. Therefore, in 2009, the International Agency for Research on Cancer (IARC) classified absorbed *Clonorchis* worms as a Class I biological carcinogen [28].

Liver fibrosis is primarily caused by chronic liver injury due to hepatitic virus infection, parasite infection, toxins, autoimmune diseases and other factors. During the liver fibrotic process, normal liver tissues are gradually replaced by accumulated ECM proteins, which are mainly produced by activated HSCs and activated portal fibroblasts [29]. The ECM is mainly composed of collagen, fibronectin and N-cadherin, among other constituents [30, 31]. In the current study, we found that with the prolongation of *C. sinensis* infection, the expression of collagen fiber and N-cadherin gradually increased, whereas the expression of desmin protein, a biomarker of HSCs, decreased (Fig. 1d–g, Fig. 2d–e). This result confirms that the activation of HSCs gradually increases with continuous parasite stimulation, which in turn leads to the production of ECM proteins.

EBI3, a subunit of IL-35 and IL-27, can cooperate with cadherin under inflammatory conditions and play an important role in promoting proper protein folding [12]. Studies have found that EBI3 can alleviates bleomycin-induced pulmonary fibrosis by suppressing DNA enrichment of STAT3. Additionally, EBI3 downregulation contributes to type I collagen overexpression in scleroderma skin [15, 16]. Our results showed that although the expression of serum EBI3 increased during the inflammatory stage of *C. sinensis* infection, it decreased significantly during the fibrogenesis phase (Fig. 2a). This finding is consistent with the result of clinical experiments that showed reduced serum EBI3 expression in patients with liver cirrhosis [13]. Similarly, we found that the expression of liver EBI3 protein remained elevated after infection but decreased in the fibrogenesis phase compared with the inflammatory stage (Fig. 2b; Additional file 1: Fig. S2A). Interestingly, the hepatic EBI3 mRNA transcript level of the infected mice was decreased at week 4 post-infection but was significantly higher than that of mice in the uninfected group at week 16 (Fig. 2c). This result suggests that the production of EBI3 is inhibited during fibrosis and that EBI3 mRNA expression may be affected by serum EBI3 level.

Through bioinformatics analysis, we found that EBI3 may regulate collagen fibril organization through modification of ECM structural constituents (Fig. 3). As it was unclear whether EBI3 exerts this effect by regulating HSC, one of the main sources of ECM, we performed an in vitro experiment in which primary HSCs were stimulated with Cs.ESP, the excretory-secretory products of *C. sinensis*. After 12 h of stimulation, rEBI3 was added to the culture system to test the effect of EBI3 on HSCs. The results showed that rEBI3 treatment inhibited the activation of HSCs induced by Cs.ESP, leading to the decrease of COL1A1, COL3A1 and ACTA2 expressions (Fig. 4d–f).

Next, we treated the mice with rEBI3 through intravenous injection during the inflammatory stage of *C. sinensis* infection to verify the in vitro results in vivo. The results showed that at week 4 post-infection, the expression of EBI3 protein and EBI3 mRNA in the liver of mice in the rEBI3 treatment group decreased significantly, while the expressions of serum EBI3 and liver desmin were significantly higher than those in the liver of mice in the infection group (Fig. 5e–g, l). This result again confirms that the expression of EBI3 mRNA is affected by the serum EBI3 level and that the increase in serum EBI3 leads to the inhibition of HSC activation. Moreover, in rEBI3-treated mice, E-cadherin was increased, whereas N-cadherin was decreased compared to that in the infected mice (Fig. 5h–i). This result is consistent with the finding that E-cadherin mRNA expression is decreased and N-cadherin mRNA is increased in biliary tract cancer [32]. All of the above results provide evidence that EBI3 can directly interact with HSCs and then inhibit the HSC production by the EMT and ECM.

EBI3 has two type of receptors: gp130 and the IL-12 receptor, beta 2 subunit (IL-12R-β2). Gp130 is expressed on all cells, while IL-12R-β2 is mostly expressed on T cells, natural killer (NK) cells, some B cells and dendritic cells (DC) [33]. Studies on IL-6, another ligand of gp130, have found that IL-6 and the IL-6Rα complex triggering association with gp130 can initiate intracellular signal transduction through the JAK/STAT signaling pathway [34, 35]. Researchers have documented that STAT3 is closely related to the occurrence and development of liver fibrosis caused by various factors [36]. Chen et al. found that the egg antigen p40 of *Schistosoma japonicum* can promote senescence in activated HSCs by activating the STAT3 pathway [37]. In the present study, we found that the expressions of gp130, JAK1, p-JAK1, STAT3 and p-STAT3 were significantly inhibited in HSCs stimulated with Cs.ESP. However, when HSCs were co-cultured with Cs.ESP and rEBI3, or when rEBI3 was injected into the tail vein of mice infected with *C. sinensis*, the expressions of these proteins were significantly increased (Figs. 4g, 5q). This result indicates that the expression of gp130 on *C. sinensis-*induced activated HSCs is suppressed, but that rEBI3 treatment could reverse this outcome, followed by activation of the JAK1/STAT3 pathway.

Cytokines play a crucial role in the development of fibrosis. IFN-γ is believed to reduce the occurrence of fibrosis, while IL-4, IL-13 and transforming growth factor-beta (TGF-β) can promote fibrosis [38–40]. In our study, we found that rEBI3 treatment led to changes in the serum levels of various cytokines 28 days after infection. Specifically, the levels of IFN-γ increased, while the levels of IL-4, TNF, IL-6 and IL-10 decreased (Fig. 6e–i). These findings suggest that rEBI3 treatment may modulate the cytokine environment and potentially affect the development of fibrosis. However, the exact mechanism by which EBI3 modulates cytokine production and affects the development of fibrosis remains unclear. Further studies are needed to fully understand the role of EBI3 in regulating cytokine production and its potential as a target for fibrosis treatment.

## Conclusions

**Fig. 7:**
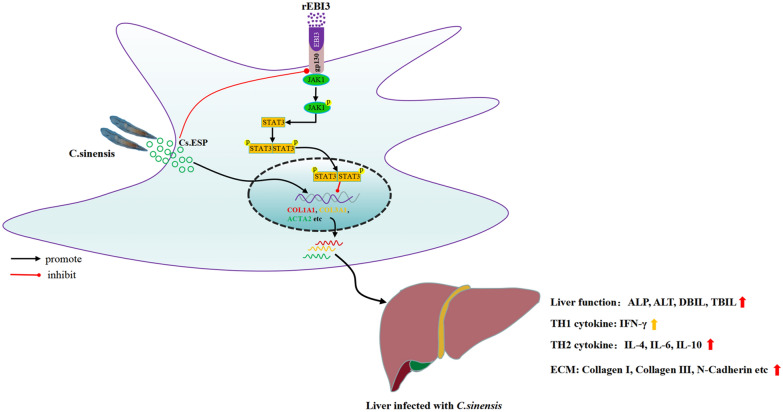
Schematic model showing that rEBI3 attenuates *C. sinensis*-induced fibrosis by inhibiting hepatic stellate cell activation through the JAK1/STAT3 pathway

In conclusion, our study revealed that Cs.ESP activates HSCs and inhibits gp130 expression, which in turn leads to increased ECM generation and eventually to fibrosis. However, the increased expression of serum EBI3 can promote the high expression of gp130 in HSCs, activating the JAK1/STAT3 pathway, which in turn inhibits the expression of ECM and leads to attenuated liver fibrosis (Fig. 7). Our data provide a new mechanism implicated in liver fibrosis and also provide a new approach for the treatment of liver fibrosis.

### Supplementary Information

**Additional file 1. Table S1**: Sequences of primers for quantitative real-time PCR. **Table S2**: Primary antibody for western blot analysis. **Fig. S1**: Relative protein expression in mice infected with *C. sinensis* at different stages, related to Fig. 2. **Fig. S2**: rEBI3 can inhibit the activation of HSC induced by Cs. ESP though the activated JAK1/STAT3 signal pathway, related to Fig. 4. **Fig. S3**: Expression of liver-related proteins in mice infected with *C. sinensis* after tail vein injection of rEBI3 (3.5 μg/100 μL) or NS (100 μL) at 4 weeks, related to Fig. 5. **Fig. S4**: Expression of liver related proteins in mice infected with *C. sinensis* after tail vein injection of rEBI3 (3.5 μg/100 μL) or NS (100 μL) at 4 weeks, related to Fig. 5q.

## Data Availability

The datasets generated and/or analysed during the current study are available from the corresponding author on reasonable request.

## Abbreviations

ACTA2: Actin alpha 2, smooth muscle
ALP: Alkaline phosphatase
ALT: Alanine aminotransferase
COL1A1: Collagen type I alpha 1
COL3A1: Collagen type III alpha 1
Cs.CA: *C. sinensis* adult crude antigen
Cs.ESP: *C. sinensis* excretory-secretory products
DBIL: Direct bilirubin
EBI3: Epstein-Barr virus-induced gene 3
ECM: Extracellular matrix
EMT: Epithelial-mesenchymal transition
GO: Gene Ontology
gp130: Beta receptor glycoprotein 130
H&E: Hematoxylin and eosin
HSC: Hepatic stellate cell
HYP: Hydroxyproline
IFN: Interleukin
IL: Interferon
JAK: Janus tyrosine kinase protein
KEGG: Kyoto Encyclopedia of Genes and Genomes
MFB: Myofibroblast
NS: Normal saline
STAT: Signal transducer and activator of transcription
TBIL: Total bilirubin
Th: T helper
TNF: Tumor necrosis factor
