# Supplementary material for: Recombinant protein EBI3 attenuates Clonorchis sinensis-induced liver fibrosis by inhibiting hepatic stellate cell activation in mice
Source: Parasit Vectors. 2023 Jul 21;16:246. doi: 10.1186/s13071-023-05863-5 (PMC10360228; doi:10.1186/s13071-023-05863-5)
Supplement: Supplementary file 1 — Additional file 1. Table S1: Sequences of primers for quantitative real-time PCR. Table S2: Primary antibody for western blot analysis. Fig. S1: Relative protein expression in mice infected with C. sinensis at different stages, related to Fig. 2. Fig. S2: rEBI3 can inhibit the activation of HSC induced by Cs. ESP though the activated JAK1/STAT3 signal pathway, related to Fig. 4. Fig. S3: Expression of liver-related proteins in mice infected with C. sinensis after tail vein injection of rEBI3 (3.5 μg/100 μL) or NS (100 μL) at 4 weeks, related to Fig. 5. Fig. S4: Expression of liver related proteins in mice infected with C. sinensis after tail vein injection of rEBI3 (3.5 μg/100 μL) or NS (100 μL) at 4 weeks, related to Fig. 5q. [file 13071_2023_5863_MOESM1_ESM.docx]

**Recombinant protein EBI3 attenuates *Clonorchis sinensis*-induced liver fibrosis by inhibiting hepatic stellate cell activation in mice**

**Lei Zhao^1^†, Jia Li^1^†, Gang Mo^1^†, Deping Cao^1^, Chun Li^1^, Guoyang Huang^1^, Liping Jiang^1^, Gen Chen^1^, Hongbing Yao^3^ and Xiaohong Peng^1,2*^**

†Lei Zhao , Jia Li and Gang Mo contributed equally to this work.

1.Guangxi University Key Laboratory of Pathogenic Biology, Guilin Medical University, Guilin, People's Republic of China.

2. Guangxi Key Laboratory of Molecular Medicine in Liver Injury and Repair, the Affiliated Hospital of Guilin Medical University, Guilin, People's Republic of China.

3. Second Affiliated Hospital of Guilin Medical University, Guilin, People's Republic of China.

*Correspondence: pxh815@163.com

**Supporting Tables**

Table S1: Sequences of primers for quantitative real-time PCR

| Gene Name | Primers (5′–3′) forward | Primers (5′–3′) reverse |
| --- | --- | --- |
| β-actin | GGCTGTATTCCCCTCCATCG | CCAGTTGGTAACAATGCCATGT |
| EBI3 | GCTCCCCTGGTTACACTGAA | TGAAGGACGTGGATCTGGTG |
| COL1A1 | AGAGCCTGAGTCAGCAGATTG | AGTAGACCTTGATGGCGTCC |
| ACTA2 | TGCTGGACTCTGGAGATGGT | ATCTCACGCTCGGCAGTAGT |
| COL3A1 | CCCACTATTATTTTGGCACAACAG | AACGGATCCTGAGTCACAGACA |
| Desmin | TATTGACCTGGAGCGCAGAA | TCATACTGAGCCCGGATGTC |
| Cdh1 | TGGTGTGGGTCAGGAAATCA | CACATGCTCAGCGTCTTCTC |
| Cdh2 | AGAACAGGGTGGACGTCATT | ACCACTGTGACTAGCCCATC |

Table S2: Primary antibody for western blot analysis

| Antibody Name | Dilutions | Manufacturer | Catalog No. | Species |
| --- | --- | --- | --- | --- |
| EBI3 (mAb) | 1:1000 | ABclonal (CHN) | A19613 | Rabbit |
| Desmin (mAb) | 1:2000 | ABclonal (CHN) | A3736 | Rabbit |
| N-Cadherin (pAb) | 1:1000 | ABclonal (CHN) | A0433 | Rabbit |
| E-Cadherin (pAb) | 1:1000 | ABclonal (CHN) | A11509 | Rabbit |
| α-SMA (mAb) | 1:500 | ABclonal ( CHN) | A17910 | Rabbit |
| Collagen Ⅰ (mAb) | 1:500 | ABcam (UK) | Ab260043 | Rabbit |
| Collagen Ⅲ (pAb) | 1:1000 | Proteintech (USA) | 22734-1-AP | Rabbit |
| gp130 (pAb) | 1:1000 | ABclonal ( CHN) | A3365 | Rabbit |
| JAK1 (mAb) | 1:1000 | ABclonal (CHN) | A11963 | Rabbit |
| p-JAK1 (pAb) | 1:4000 | ABclonal (CHN) | AP0530 | Rabbit |
| STAT3 (pAb) | 1:2000 | ABclonal (CHN) | A1192 | Rabbit |
| p-STAT3 (pAb) | 1:2000 | ABclonal (CHN) | AP0530 | Rabbit |
| β-Tubulin (mAb) | 1:2000 | ABclonal (CHN) | AC021 | Rabbit |
| β-actin (pAb) | 1:1000 | Proteintech (USA) | 20536-1-AP | Rabbit |
| GAPDH (pAb) | 1:1000 | Affinity (CHN) | AF7021 | Rabbit |

**Supplementary Figures**

**
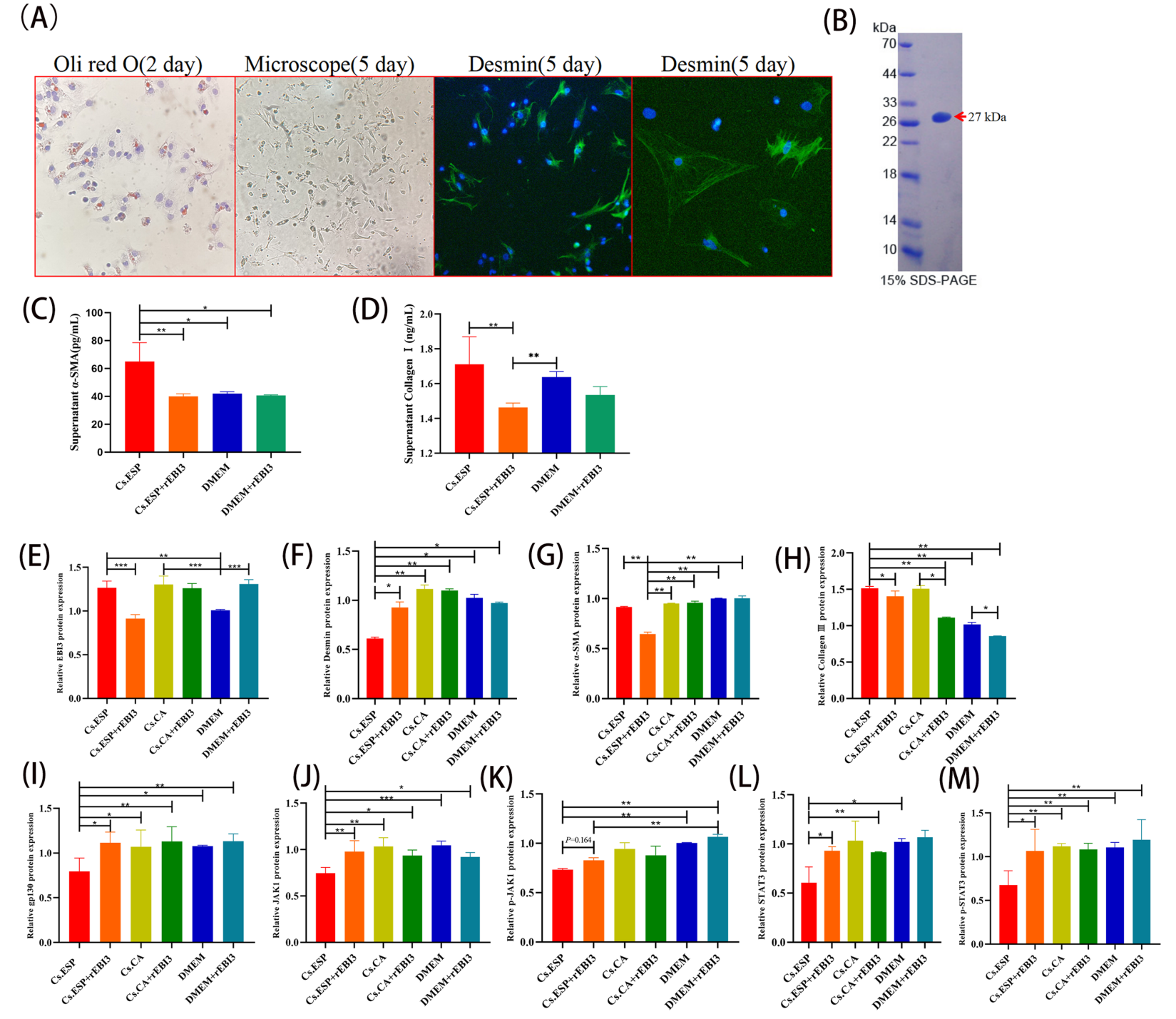
Supplementary Figures 1**

**Fig. S1. rEBI3 can inhibit the activation of HSC induced by Cs. ESP though the activated JAK1/STAT3 signal pathway, related to Fig 4. (A)** Staining of hepatic stellate cells **(B)** SDS-PAGE diagram of rEBI3. **(C-D)** α-SMA, Collagen Ⅰ content of HSC co-cultured supernatant. **(E-H)** The relative protein contents of EBI3, Desmin, α-SMA, and Collagen Ⅲ. **(I-M)** The signal pathway relative protein contents of **(I)** gp130 **(J)** JAK1 **(K)** p-JAK1 **(L)** STAT3 **(M)** p-STAT3 are shown. The data were presented as the mean ±SD. Data were compared with the one-way analysis of variance (ANOVA), ^*^*p* <0.05, ^**^*p* <0.01, ^***^*p* <0.001, (The data represents one of two independed experiments, and each experimental group has made 3 multiple holes.).

**Supplementary Figures 2**

**
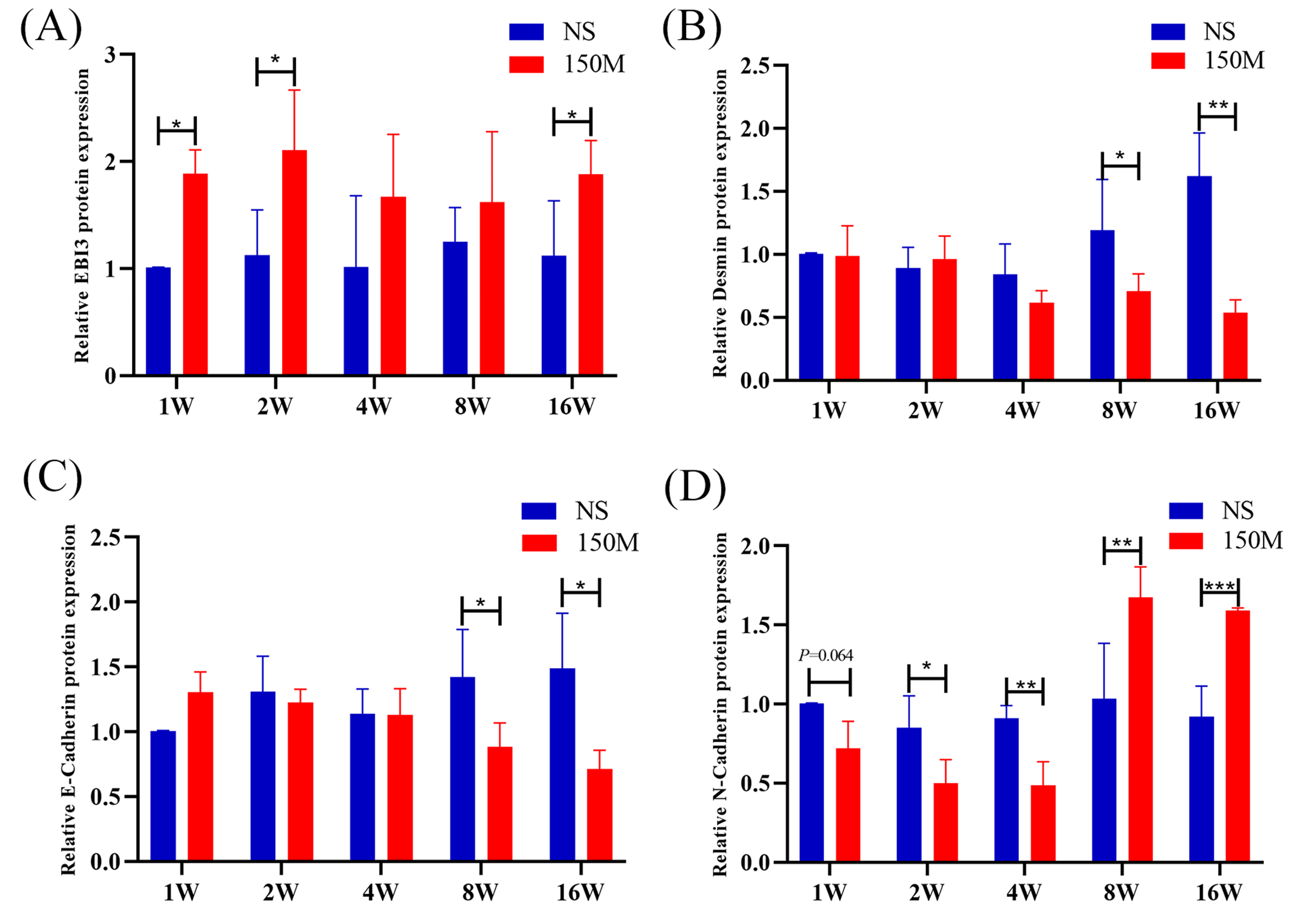
**

**Fig. S2. Relative protein expression in mice infected with *C. sinensis* at different stages, related to Fig 2.** Total liver proteins were extracted and subjected to western blot analysis with indicated antibodies. The relative protein contents **(A)** EBI3 **(B)** Desmin **(C)** E-Cadherin **(D)** N-Cadherin were shown. The data were presented as the mean±SD. Data were compared with the unpaired *t* test(two tailed), **p* <0.05, ***p* <0.01, ****p* <0.001. (The data represents one of two independed experiments, 6 mice in each group and correspond to mean±SD).

**Supplementary Figures 3**

**
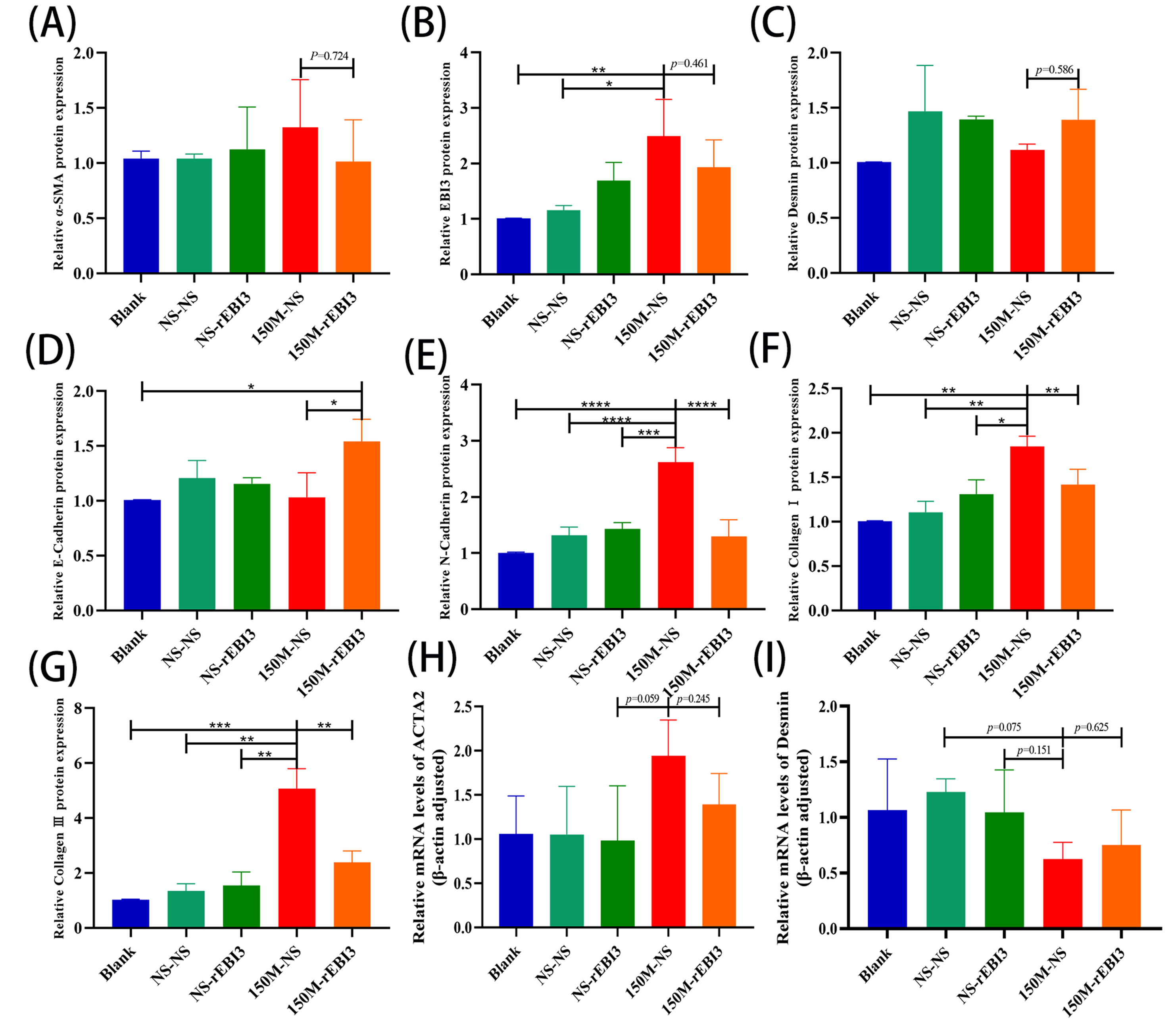
**

**Fig. S3. Expression of liver-related proteins in mice infected with *C. sinensis* after tail vein injection of rEBI3 (3.5 μg/100 μL) or NS (100 μL) at 4 weeks, related to Fig 5.** Total liver protein and RNA were extracted and subjected to western blot or RT-qPCR analysis with indicated antibodies and primers. The relative protein content **(A)** α-SMA **(B)** EBI3 **(C)** Desmin **(D)** E-Cadherin **(E)** N-Cadherin **(F)** Collagen Ⅰ **(G)** Collagen Ⅲ and mRNA expression **(H)** ACTA2 **(I)** Desmin are shown. The data are presented as the mean±SD. Data were compared with the one-way analysis of variance (ANOVA), **p* <0.05, ***p* <0.01, ****p* <0.001, (The data represents 6 individually analyzed mice in each group and correspond to mean±SD).

**Supplementary Figures 4**

**
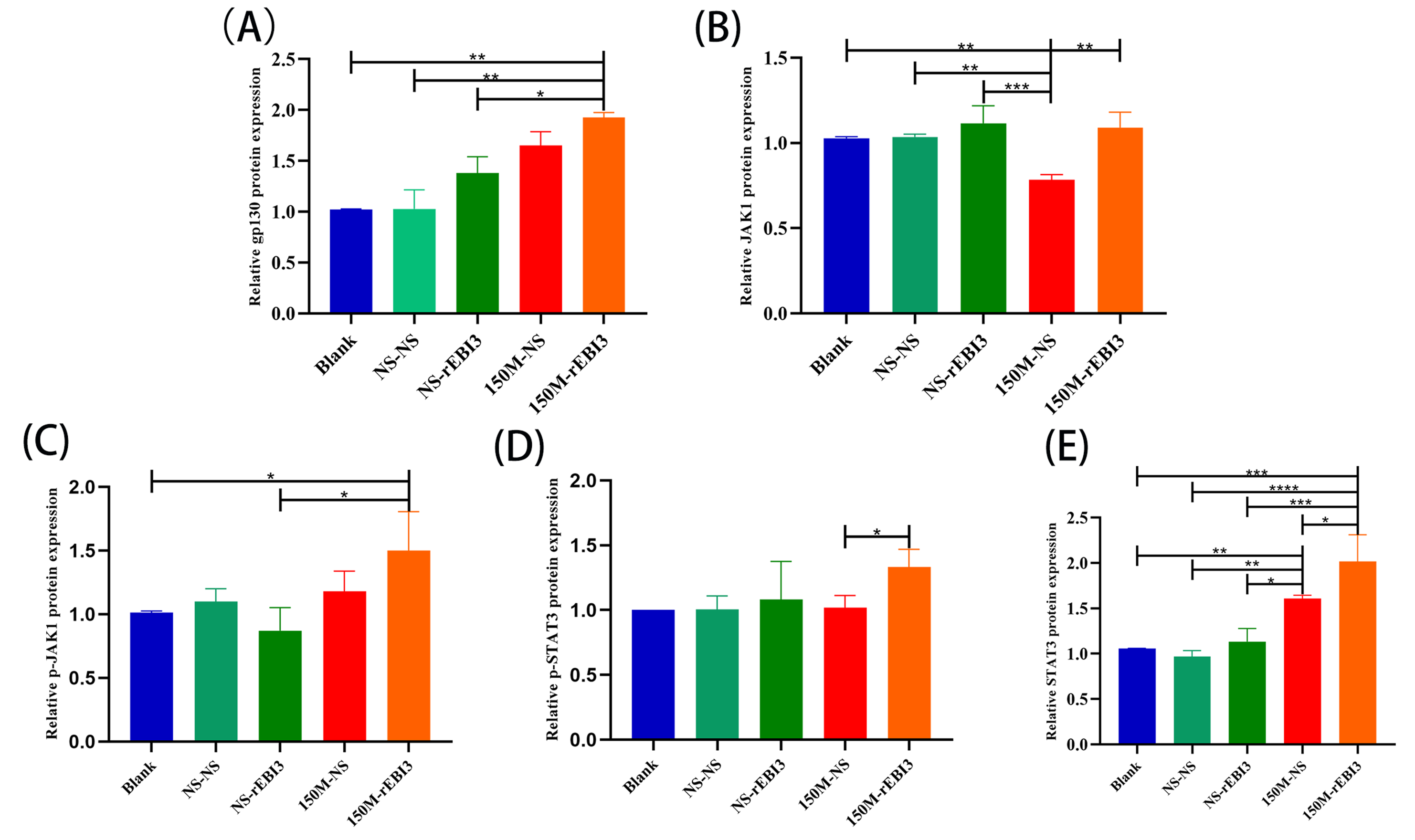
**

**Fig. S4. Expression of liver related proteins in mice infected with *C. sinensis* after tail vein injection of rEBI3 (3.5 μg/100μL) or NS (100 μL) at 4 weeks, related to Fig 5Q.** Total liver proteins were extracted and subjected to western blot analysis with indicated antibodies. The relative protein contents **(A)** gp130 **(B)** JAK1 **(C)** p-JAK1 **(D)** p-STAT3 **(E)** STAT3 were shown. The data were presented as the mean±SD. Data were compared with the one-way analysis of variance (ANOVA), **p* <0.05, ***p* <0.01, ****p* <0.001, (The data represents 6 individually analyzed mice in each group and correspond to mean±SD).
